# Ironing out the Details: Exploring the Role of Iron and Heme in Blood-Sucking Arthropods

**DOI:** 10.3389/fphys.2017.01134

**Published:** 2018-01-17

**Authors:** Shavonn R. Whiten, Heather Eggleston, Zach N. Adelman

**Affiliations:** ^1^Department of Entomology, Texas A&M University, College Station, TX, United States; ^2^Genetics Graduate Program, Texas A&M University, College Station, TX, United States

**Keywords:** iron, heme, arthropod, mosquito, tick, bloodfeeding, peritrophic matrix, transporter

## Abstract

Heme and iron are essential molecules for many physiological processes and yet have the ability to cause oxidative damage such as lipid peroxidation, protein degradation, and ultimately cell death if not controlled. Blood-sucking arthropods have evolved diverse methods to protect themselves against iron/heme-related damage, as the act of bloodfeeding itself is high risk, high reward process. Protective mechanisms in medically important arthropods include the midgut peritrophic matrix in mosquitoes, heme aggregation into the crystalline structure hemozoin in kissing bugs and hemosomes in ticks. Once heme and iron pass these protective mechanisms they are presumed to enter the midgut epithelial cells via membrane-bound transporters, though relatively few iron or heme transporters have been identified in bloodsucking arthropods. Upon iron entry into midgut epithelial cells, ferritin serves as the universal storage protein and transport for dietary iron in many organisms including arthropods. In addition to its role as a nutrient, heme is also an important signaling molecule in the midgut epithelial cells for many physiological processes including vitellogenesis. This review article will summarize recent advancements in heme/iron uptake, detoxification and exportation in bloodfeeding arthropods. While initial strides have been made at ironing out the role of dietary iron and heme in arthropods, much still remains to be discovered as these molecules may serve as novel targets for the control of many arthropod pests.

## Introduction: evolution of arthropod hematophagy

Estimates suggest there are more than 1 million insect and arachnid species inhabiting planet Earth, approximately 14,000 of which have adapted the ability to feed on vertebrate blood (Graça-Souza et al., 2006). Commonly known as hematophagy, this adaptation has arisen independently many times over the course of evolution (Graça-Souza et al., 2006). However, certain features are shared among the ancestors of bloodfeeding arthropods. Traditionally accepted ancestral pre-conditions to bloodfeeding include: (1) living in close proximity to vertebrates, (2) specialized feeding on skin remains, dung or fluids from animal carcasses, and (3) specialized mouthparts for piercing and cutting (Graça-Souza et al., 2006). It is quite interesting that hematophagous arthropods can consume anywhere from 2 to 100x their normal body weight during a single blood meal. Subsequently, this blood meal, comprised largely of proteins, is digested by enzymes secreted from midgut epithelial cells (Barillas-Mury and Wells, 1993; Jiang et al., 1997; Edwards et al., 2000; Brackney et al., 2010). The amino acids that result from blood meal protein digestion are used for lipid, carbohydrate, and egg protein synthesis (Marquardt and Kondratieff, 2005). While the bloodfeeding process has obvious nutritional advantages for hematophagous arthropods, the digestive process releases the pro-oxidant molecules heme and iron in potentially toxic quantities. In response to this challenge, bloodfeeding arthropods have evolved a number of strategies to limit oxidative damage during blood digestion. This review seeks to compile and compare existing vertebrate and invertebrate literature as it pertains to iron and heme processing, with particular interest in applying knowledge gained in other systems to hematophagous arthropods.

## Protective adaptations against oxidative stress after a blood meal: antioxidants

Over time, hematophagous arthropods have honed their antioxidant systems to avoid oxidative stress (Felton and Summers, 1995; Champion and Xu, 2017). Oxidative stress has traditionally been defined as the disturbance in balance between the production of reactive oxygen species (ROSs) and antioxidant defenses (Felton and Summers, 1995; Betteridge, 2000; Chaitanya et al., 2016), which may result in the oxidation of nucleic acids (Ryter and Tyrrell, 2000), lipids (Tappel, 1955; Vincent et al., 1988), and proteins (Aft and Mueller, 1984; Vincent et al., 1988). However, all cells and organisms naturally produce ROSs (Chaitanya et al., 2016). For example, prior to a blood meal, the mosquito *Aedes aegypti*, the most important vector of arboviruses worldwide, generate ROSs for the control of intestinal microbiota proliferation (Oliveira et al., 2011). Oliveira and Oliveira (2002) hypothesized that a decrease in mitochondrial ROSs may be necessary to avoid their interaction with the pro-oxidant products of blood meal digestion, heme and iron (Oliveira and Oliveira, 2002). This was confirmed by Gonçalves et al. (2009), who reported that bloodfeeding promoted the fusion of *Ae. aegypti* flight muscle mitochondria, which decreased the generation of ROSs (Gonçalves et al., 2009). Interestingly, mitochondrial decreases in H_2_O_2_ followed the digestion process, with mitochondrial function returning to pre-blood meal levels upon the completion of digestion (Gonçalves et al., 2009). Oliveira et al. (2011) found that this decrease in ROSs is triggered by heme and no other blood component (Oliveira et al., 2011). To further reduce ROSs generated post bloodfeeding and avoid a milieu prone to oxidative stress, *Ae. aegypti* and other hematophagous arthropods have deployed a number of defenses into the gut lumen and hemolymph (extracellular) as well as in the cytoplasm of midgut epithelial cells or other tissues in the body (intracellular).

### Intracellular antioxidants

Intracellularly, Cu, Zn, and Mn superoxide dismutases (SOD) catalyze the conversion of superoxide anions into hydrogen peroxide. Subsequently, catalase (CAT) and general peroxidases (POD) are responsible for detoxifying hydrogen peroxide (Felton and Summers, 1995). More specifically, catalase catalyzes the dismutation of hydrogen peroxide to oxygen and water. Paes et al. (2001) found that when compared to other tissues, SOD and CAT activity were highest in the adult female midgut of the kissing bug, *Rhodnius prolixus*. Likewise, subsequent inhibition of CAT resulted in increased H_2_O_2_ in midgut extracts (Paes et al., 2001). To further test the individual and collective roles of CAT and glutathione (GSH), each antioxidant was individually and collectively inhibited. For GSH and CAT, there was a 4- and 2-fold increase in H_2_O_2_ content in *R. prolixus* midgut extracts, respectively. Interestingly, when GSH and CAT synthesis were inhibited simultaneously, the authors saw a tremendous increase in midgut extract H_2_O_2_ content, confirming the importance of both antioxidants with regards to H_2_O_2_ control after a blood meal (Paes et al., 2001). In a more recent study, through gene expression comparisons, Oliveira et al. (2017) found that mRNA levels for the antioxidant enzyme CAT increased 6-fold at 24 and 36 h after a blood meal and decreased to values equivalent to sugar fed mosquitoes at the end of digestion (72 h). RNAi-mediated knockdown of CAT resulted in reduced oviposition and lifespan when adult female *Aedes aegypti* were challenged with the pro-oxidant H_2_O_2_ (Oliveira et al., 2017). This reduced fecundity as a result of RNAi mediated knockdown of CAT was also demonstrated in adult female *Anopheles gambiae, one* of the primary mosquito vectors of malaria parasites (DeJong et al., 2007). Interestingly, this study found that a single serine to tryptophan polymorphism resulted in decreased CAT activity and stability (DeJong et al., 2007). The antioxidant role of CAT in ovaries has also been documented in other Dipterans besides mosquitoes, as ovarian CAT was found to accumulate in developing oocytes of the bloodsucking sand fly, *Lutzomyia longipalpis*, 12–48 h after bloodfeeding (Diaz-Albiter et al., 2011). Beyond SOD and CAT, glutathione S-transferase (GST) catalyzes the conjugation between glutathione and other molecules (Freitas et al., 2007). Through competitive enzymatic-based assays and assays measuring changes in intrinsic fluorescence, *Ae. aegypti* recombinant GSTX2-2 was the first mosquito GST found to *in vitro* bind heme (Lumjuan et al., 2007). However, further studies are needed to determine antioxidant functionality for GSTX2-2 after a blood meal. In the cattle tick, *Rhipicephalus* (*Boophilus*) *microplus*, GST activity was confirmed as antioxidant in the eggs and larvae of engorged females. More specifically, GST enzymatic activity significantly increased and O_2_ consumption progressively increased during embryonic development and eventually peaked at hatch (Freitas et al., 2007). These authors reported a strong correlation between O_2_ consumption and GST activity, as well as a positive correlation for the antioxidants CAT and GSH with GST in *R. microplus* eggs and larvae. These results suggest that in addition to the antioxidant parameters, which allow for avoidance of oxidative stress in the midgut after a blood meal, increased oxidative stress can be associated with embryogenesis and aging (Freitas et al., 2007).

### Extracellular antioxidants

While certain antioxidant molecules work in concert to intracellularly reduce oxidative stress, here we highlight recent advancements regarding extracellular antioxidants. Recently, Lima et al. (2012) suggested a new role for xanthurenic acid (XA) as a heme and iron chelator. XA is product of tryptophan degradation through the kynurenine pathway. Traditionally, in insects, the kynurenine pathway is associated with eye pigment formation. However, reverse phase HPLC and mass spectrometry were used to identify xanthurenic acid (XA) as a component of *Ae. aegypti* midgut homogenates after a blood meal (ABM) (Lima et al., 2012). At 24 h ABM, XA reached maximum levels. This is also the time of peak of blood meal hemoglobin digestion, when large amounts of heme and iron are present in the midgut lumen. Both heme and iron-induced lipid peroxidation were inhibited by XA through the *in vitro* binding of XA to both heme and iron, suggesting an antioxidant role for XA in the *Ae. aegypti* midgut after a blood meal. Similarly, an antioxidant function has been demonstrated for a 15 kDa heme-binding protein (RHBP) in *R. prolixus* (Dansa-Petretski et al., 1995). Given that RHBP is a hemolymph-localized heme-binding protein, it will be further discussed in this context later in our review. However, using ^32^P labeling of fat bodies (site of lipid production), Dansa-Petretski et al. (1995) provided evidence that RHBP serves as an antioxidant, allowing for proper functioning of lipophorin as a lipid shuttle from the fat bodies to other organs. Heme negatively affected the functionality of lipophorin, but functionality returned to normal levels when RHBP was present (Dansa-Petretski et al., 1995). More specifically, RHBP inhibited the heme-induced reduction of lipophorin ability to transfer phospholipids to the ovaries (Dansa-Petretski et al., 1995).

The above studies highlight various antioxidant molecules deployed by some bloodfeeding arthropods. Undoubtedly, many additional mechanisms remain to be uncovered, particularly in those bloodfeeding species that do not receive as much attention, such as lice, fleas and bed bugs. We note that the subject of redox homeostasis is even more complicated in context of the microbiota of bloodfeeding arthropods. This topic was the subject of a recent review (Champion and Xu, 2017), and thus will not be discussed further.

## Protective adaptations against oxidative stress after a blood meal: specialized midgut defense mechanisms

Hematophagous arthropods have evolved specialized defense mechanisms to avoid heme toxicity during blood meal digestion such as the extracellular double plasma membrane structure, perimicrovillar membranes (PPM) (Lane and Harrison, 1979; Silva et al., 2004; Gutiérrez-Cabrera et al., 2015) which cover the midgut epithelia and facilitate generation of the heme crystal, hemozoin in the kissing bug (Oliveira et al., 1999, 2000, 2005; Pagola et al., 2000; Silva et al., 2004, 2007), hemosomes in the cattle tick (Braz et al., 1999; Lara et al., 2003), and Type I peritrophic matrix formation in larval and nymphal *Ixodes scapularis* ticks and adult Dipteran mosquitoes (except Tsetse adults which form Type II PM) (Peters, 1992; Narasimhan et al., 2014; Rose et al., 2014). Interestingly, all of the above-mentioned structures function in heme detoxification through various specialized forms of aggregation or crystalization (Graça-Souza et al., 2006). Below we have highlighted recent advancements in knowledge regarding each of these structures, but refer the reader to Graça-Souza et al. (2006) for a more detailed review of earlier literature (Graça-Souza et al., 2006).

In the kissing bug *R. prolixus*, transmission electron micrographs of the midgut showed large aggregates of crystalline heme during blood meal digestion (Oliveira et al., 1999, 2000). Interestingly, heme crystallization was also found in three other triatomine insects, *Triatoma infestans, Dipetalogaster maximus* and *Panstrongylus megistus* (Oliveira et al., 2007). Further investigation identified the perimicrovillar membranes, extracellular double plasma membranes which line the midgut epithelium, as necessary for heme crystallization into hemozoin for *R. prolixus, T. infestans, D. maximus*, and *P. megistus* (Oliveira et al., 2005, 2007; Silva et al., 2007). Specifically, lipid and protein constituents of the perimicrovillar membrane were necessary for proper hemozoin formation (Silva et al., 2007; Stiebler et al., 2010, 2014). Hemozoin formation assays and RNAi mediated knock-down of α-glucosidase provided evidence supporting the role of α-glucosidase in the nucleation step of hemozoin formation (Mury et al., 2009). α-glucosidase is a membrane-bound enzyme found in the midgut of *R. prolixus* with maximal activity 7–9 days post blood meal (PBM) (Silva et al., 2007). Interestingly, aggregated hemozoin also reached maximum levels at 7 days PBM (Silva et al., 2007). More recently, through kinetic analysis of hemozoin formation induced by the perimicrovillar membranes, Stiebler et al. (2010) identified three different stages of hemozoin formation in *R. prolixus*. Through Mössbauer spectroscopy, these authors provided evidence that at least 97% of all iron present in the midguts of *R. prolixus* 4 days post blood meal was in the form of hemozoin (Stiebler et al., 2010). Follow-up experiments provided evidence that lipids isolated from the perimicrovillar membranes are efficient catalysts of hemozoin formation *in vitro* (Stiebler et al., 2010, 2014). More specifically, *in vitro* experiments with two commercial phospholipids, unsaturated 1,2-dilinoleoyl-sn-glycero-3-phosphoethanolamine (uPE) and to a lesser extent unsaturated 1,2-dilinoleoyl-sn-glycero-3-phosphocholine (uPC) produced brick shaped and blunt ended β-hematin crystals similar to those produced by plasma-fed or blood-fed *R. prolixus* midgut lipids (Stiebler et al., 2014). This suggests that uPE and uPC (to lesser extent) play integral roles in the determination of crystal morphology for hemozoin in the midgut of *R. prolixus*. This is in agreement with previous findings, which reported 1,2-dilinoleoyl-sn-glycero-3-phosphoethanolamine as the major phospholipid synthesized by *R. prolixus* midgut epithelium (Bittencourt-Cunha et al., 2013). A similar involvement of lipids in hemozoin formation has been well documented in the parasites *Plasmodium* and *Schistosoma mansoni* (blood fluke) (Oliveira et al., 2004; Corrêa Soares et al., 2007; Stiebler et al., 2010, 2014). Taken together, these results emphasize the importance of looking beyond protein components for the mechanistic basis of important biochemical reactions in bloodmeal detoxification.

*R. microplus* are unable to endogenously synthesize heme (Braz et al., 1999). For the first 7 days after a blood meal, hemoglobin-derived heme is directly absorbed for vitellogenesis (Lara et al., 2003). During this time, the largest amount of heme is concentrated at the basal lamina side of midgut digestive cells (closest to hemocoel) (Lara et al., 2003). This contrasts with other hematophagous arthropods where digestion occurs in the midgut lumen. After the first 7 days PBM, the amount of heme absorption decreases and heme concentrates in midgut digestive cell organelles that specialize in heme sequestration, termed hemosomes (Lara et al., 2003). By the end of blood meal digestion, the mass of an individual hemosome was reported to be 90% heme (Lara et al., 2003). More recently, Lara et al. (2015) proposed a detailed model for heme movement in digestive cells of *R. microplus*. However, there remain unanswered questions regarding movement of heme from digestive vesicles to the intracellular hemosomes (Lara et al., 2015).

While digestive cell hemosomes (*R. microplus*) and heme crystal hemozoins in association with a perimicrovillar membrane (*R. prolixus*) have been documented as protective mechanisms against oxidative stress, *I. scapularis* ticks (vector of *Borrelia burgdorferi*) (Narasimhan et al., 2014) and most adult Dipterans (biting flies and mosquitoes) produce a peritrophic matrix in response to bloodfeeding (Dimopoulos et al., 1998; Shen and Jacobs-Lorena, 1998; Morlais and Severson, 2001; Shao et al., 2001, 2005; Hao and Aksoy, 2002; Devenport et al., 2005, 2006; Ramalho-Ortigão et al., 2007; Jochim et al., 2008; Dinglasan et al., 2009; Narasimhan et al., 2014; Rose et al., 2014).

**Figure 1:**
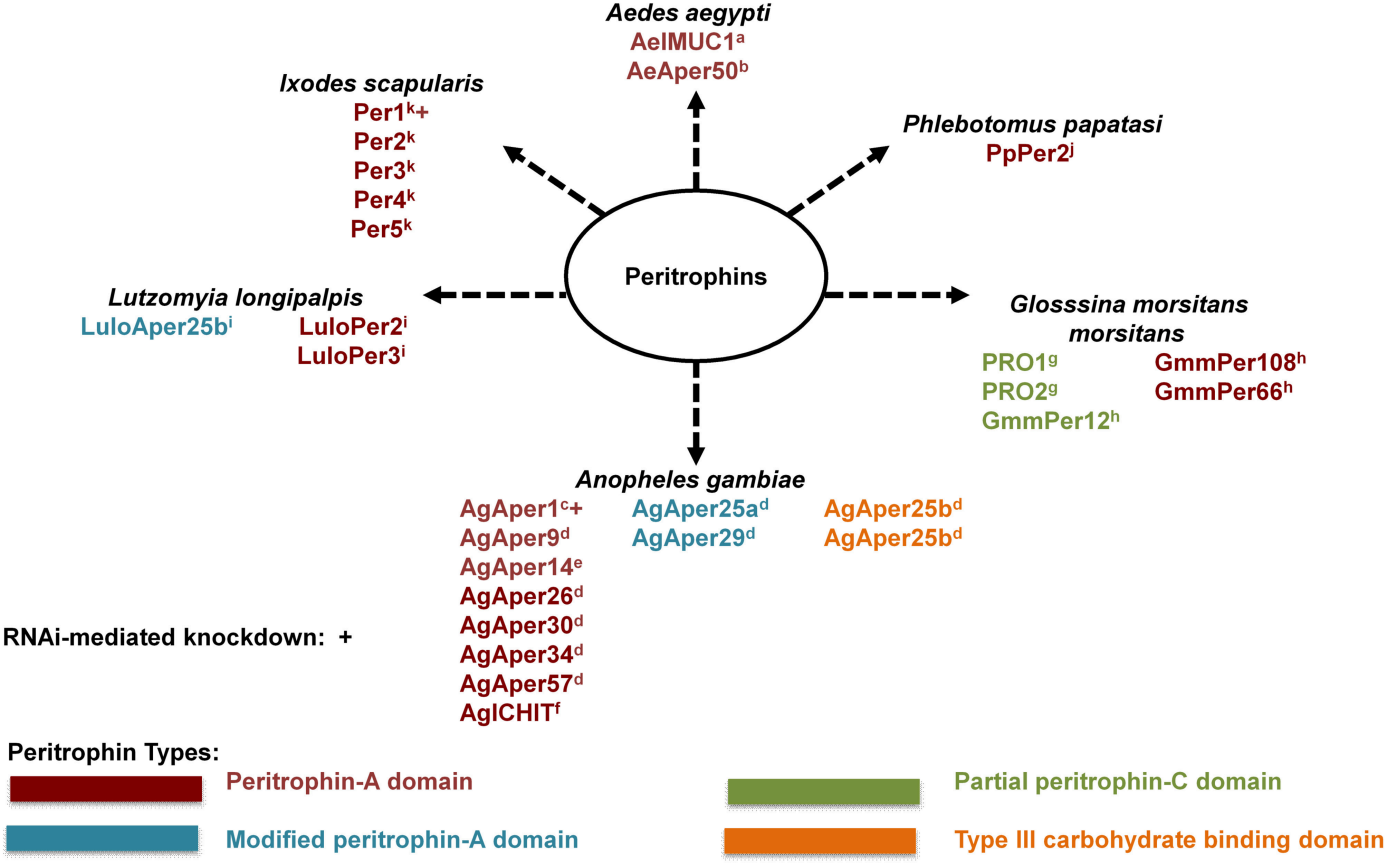
Known hematophagous arthropod peritrophic matrix peritrophins. Peritrophic matrix peritrophin classification according to their chitin-binding domains (CBDs). Peritrophin-A domain (PAD) is the most common type of CBD with six interspersed cysteine residues. Modified peritrophin-A domains (mPADs) have two additional cysteine residues. Peritrophin-C domain (PCD) contains 10 interspersed cysteine residues, and typically 121–122 amino acid residues long. Partial PCDs contain 67–70 amino acids. Type III carbohydrate binding domains (CBDIII) contains 10 interspersed cysteine residues, similar to PCD, this domain is typically 110–117 amino acid residues long. ^a^Morlais and Severson, 2001; ^b^Shao et al., 2005; ^c^Shen and Jacobs-Lorena, 1998; ^d^Dinglasan et al., 2009; ^e^Devenport et al., 2005; ^f^Dimopoulos et al., 1998; ^g^Hao and Aksoy, 2002; ^h^Rose et al., 2014; ^i^Jochim et al., 2008; ^j^Ramalho-Ortigão et al., 2007; ^k^Narasimhan et al., 2014.

The peritrophic matrix (PM) is a semipermeable extracellular layer, which lines the midgut of most invertebrates (Peters, 1992; Tellam et al., 1999). The peritrophic matrix is classified as Type I or Type II based on location of synthesis within the insect midgut (Peters, 1992). The Type I peritrophic matrix is completely synthesized by midgut epithelial cells (Tellam et al., 1999). In contrast, the Type II peritrophic matrix is constitutively secreted by the cardia, a specialized organ found between the foregut and midgut (Stohler, 1961; Moskalyk et al., 1996; Tellam et al., 1999). Type I PM formation is induced by midgut distension (Freyvogel and Jaquet, 1965; Richards and Richards, 1977) and it is comprised of proteins, proteoglycans, and chitin fibrils (Shao et al., 2001; Pascoa et al., 2002). Peritrophic matrix proteins are integral components of the PM, and are commonly referred to as peritrophins (Tellam et al., 1999). Peritrophins are characterized by the presence of a secretory signal peptide, multiple chitin-binding domains containing cysteine-proline dipeptides, and intervening mucin-like domains rich in proline, serine, and threonine (Tellam et al., 1999). The multiple chitin-binding domains of peritrophic matrix peritrophins function as cross-linkers for chitin fibrils, thereby providing structure and support for the peritrophic matrix (Schorderet et al., 1998; Shen and Jacobs-Lorena, 1998). In addition, PM peritrophins contain aromatic amino acid residues that facilitate binding with N-acetyl-glucosamine of the chitin fibrils (Toprak et al., 2010). Several Type I and Type II PM peritrophins have been identified and characterized from hematophagous insects (Figure 1).

Peritrophic matrix peritrophins can be further classified according to their chitin-binding domains (CBDs). Each CBD is comprised of cysteine residues that allow for disulfide bonds within the CBD, and the number of disulfide bonds within a CBD can range from three to five (Tellam et al., 1999; Toprak et al., 2010). The Peritrophin-A domain (PAD) is the most common type of CBD, and contains six interspersed cysteine residues (Toprak et al., 2010). Typically, an individual PAD contains 48–57 amino acid residues. However, the exact PAD sequence arrangement has been found to vary for different insect orders (Toprak et al., 2010), and as seen in *Anopheles gambiae* there are cases of modified peritrophin-A domains (mPADs). The mPAD of AgAper25a and AgAper29 are characterized as having two additional cysteine residues, however, these two cysteine residues are not considered significant (Toprak et al., 2010).

While less common, CBDs can also be classified as Peritrophin-B domain (PBD) or Peritrophin-C domain (PCD) according to the number of cysteine residues. An individual PBD contains eight interspersed cysteine amino acid residues, and is 81–88 residues long (Toprak et al., 2010). In contrast, an individual PCD contains 10 interspersed cysteine residues, and is typically 121–122 amino acid residues long (Toprak et al., 2010). As seen in the Tsetse (*Glosssina morsitans morsitans*), there are partial PCDs that contain 67–70 amino acids (Hao and Aksoy, 2002; Rose et al., 2014). In addition, as seen in *An. gambiae* AgAper25b, peritrophins can contain a Type III carbohydrate binding domains (CBDIII). While the CBDIII contains 10 interspersed cysteine residues (similar to PCD), this domain is typically 110–117 amino acid residues long.

In addition to binding chitin, one peritrophin has been reported to bind heme. *Ae. aegypti* intestinal mucin 1 (AeIMUCI) is a 275-amino acid glycoprotein that contains three chitin-binding domains and a mucin domain between CBD 1 and CBD 2 (Rayms-Keller et al., 2000; Devenport et al., 2006). Rayms-Keller et al. (2000) first identified *Ae*IMUC1 RNA in metal exposed *Ae. aegypti* mosquito larvae, metal fed adult females and blood-fed adult females (Rayms-Keller et al., 2000). Devenport et al. (2006), confirmed chitin and heme binding for AeIMUC1. Through deletion analysis using recombinant proteins, they also determined that chitin-binding and heme-binding functions are associated with the 3 CBDs of AeIMUC1 (Devenport et al., 2006). Most importantly, they confirmed AeIMUC1 as an integral PM peritrophin associated with the PM 12 to 24 h post bloodfeeding (Devenport et al., 2006). Taken together, results from previous AeIMUC1 studies suggest this protein is important for PM structural integrity (Devenport et al., 2006) and blood meal heme detoxification (Rayms-Keller et al., 2000; Devenport et al., 2006). However, studies suggest there are potentially more *Ae. aegypti* midgut peritrophic matrix peritrophins (Moskalyk et al., 1996; Pascoa et al., 2002).

With advancements in technology such as Multidimensional protein identification technology (MudPIT) (Schirmer et al., 2009), the total protein composition of tissues such as the PM can more readily be explored/discovered. For example, a similar approach to MudPIT was utilized by Dinglasan et al. (2009) to determine PM protein composition for *An. gambiae*. By utilizing an artificial protein-free meal enriched with latex beads for midgut distention and subsequent PM formation, Dinglasan et al. (2009) collected more than 750 PMs for sequential extraction, digestion and identification. In total, 209 PM and PM-associated proteins were identified via a mass spectrometry-based proteomic analysis. While the largest majority of identified proteins were associated with peptidase activity, nine PM proteins isolated in this proteomic analysis contained a secretory signal peptide and one or more chitin binding domains, and therefore took the total list of identified peritrophins from 3 to 12 in *An. gambiae*. Dinglasan et al. (2009) also proposed a model which detailed *An. gambiae* PM formation and putative interactions among the various types of proteins identified in their study (Dinglasan et al., 2009).

Given the unprecedented level of exploration and discovery of *An. gambiae* PM protein composition, and the relation of the Dinglasan et al. (2009) results to *Ae. aegypti*, another important disease vector, this methodology should be further explored to determine PM protein composition for other hematophagous arthropods of medical importance. To date, only one study with the tsetse fly (*Glossina morsitans morsitans*) has utilized a similar methodology (Rose et al., 2014). Interestingly, this study utilized teneral (unfed) male tsetse flies to determine PM protein composition, as only two PM proteins had been identified previously, but were poorly characterized (Hao and Aksoy, 2002; Rose et al., 2014). Given that adult tsetse flies produce a Type II PM, it is constitutively expressed. Therefore, the requirement of blood meal or artificial meal induction, as seen in Type I PM formation, was found not necessary for PM protein composition analyses. To maximize the number of isolated proteins, the authors conducted in-gel and in-solution tryptic digests using 150 PMs. While their in-gel analysis yielded two highly visible bands at 26 and 21 kDa (identified as midgut trypsin), the most abundant and frequent protein hit was for a novel PM protein, GmmPer6 (Rose et al., 2014). As expected, the in-solution analysis yielded substantially more proteins (minimum of 195). Based on putative function, the largest majority of isolated proteins were associated with oxidation/reduction (17%). In total, five PM peritrophins were isolated in this study (two known and three novel). As in the in-gel analysis, GmmPer66 was one of the most abundant proteins isolate din the in-solution analysis with two other novel PM peritrophins isolated (GmmPer12 and GmmPer108) (Rose et al., 2014).

Despite the identification of these structural components, few studies have utilized reverse genetic approaches such as RNAi mediated knockdown to determine physiological importance of individual peritrophins. *I. scapularis* nymphs were administered dsRNA targeting *peritrophin-1* through their anal pore and subsequently allowed to feed until repletion on *B. burgdorferi*-infected C3H/HeN mice. While peritrophin-1 knockdown nymphs engorged at a comparable rate to control nymphs, knockdown of peritrophin-1 resulted in decreased thickness of the PM and compromised structural integrity (Narasimhan et al., 2014). Interestingly, knockdown of peritrophin-1 was associated with decreased *B. burgdorferi* adherence/attachment to midgut epithelial cells. A similar effect was seen via RNAi-mediated knockdown of the signal transducer and activator of transcription (*stat)* gene which encodes STAT, the cytosolic component of the Janus kinase (JAK)/STAT pathway (Narasimhan et al., 2014), suggesting a modulatory role of STAT in peritritrophin-1 expression, and role of the intact PM in *B. burgdorferi* spirochetes adherence to midgut epithelium. RNAi-mediated knockdown of *An. gambiae* AgAper1 (most abundant CBD protein from Dinglasan et al., 2009) was used to determine its role in midgut epithelium response to microbiota in adult *Anopheles coluzzii* (Rodgers et al., 2017). Interestingly, knockdown of AgAper1 resulted in an increased immune response and translocation of bacteria from family *Enterobacteriaceae* into the body cavity. Therefore, these findings suggest that the PM of *An. coluzzii* serves as a barrier which blocks or limits dissemination of certain bacteria throughout the mosquito body (Rodgers et al., 2017).

Despite the highly successful strategies deployed by bloodfeeding arthropods to sequester heme during the process of blood digestion, heme and molecular iron are also critical nutrients that must be absorbed during digestion and transported throughout the body. In the following sections, we discuss recent progress on understanding heme and iron transport across cell membranes, as well as trafficking, packaging, signaling, and ultimate deposition into the ovaries for oogenesis, in order to systematically track the fate of blood meal heme and iron in medically important hematophagous arthropods.

## Heme uptake across cell membranes

Despite its importance as a nutrient and signaling molecule (Hooda et al., 2014; Bottino-Rojas et al., 2015), surprisingly little is known about heme transport/uptake in arthropods. New developments in this area would seem to be promising targets, particularly since this subject has been explored in many other organisms including mammals, fish, yeast and worms, as moving heme from either its point of synthesis (mitochondria) or from the diet (extracellular space) to the cell cytoplasm involves crossing membranes.

The feline leukemia virus subgroup C receptor (FLVCR1) heme transporter has been characterized in both humans and mice (Quigley et al., 2004; Byon et al., 2013; Vinchi et al., 2014; Philip et al., 2015). Flvcr1a is a 12 transmembrane (TM) domain protein while Flcvr1b is a 6 TM domain protein which is thought to homo/heterodimerize to form a functional transporter. Recently, Mercurio et al. (2015) found that Flvcr1a and Flvcr1b were both required for development of committed erythroid progenitors with Flvr1a exporting heme through the plasma membrane into the extracellular space while Flvr1b exports heme into the mitochondria (Mercurio et al., 2015). In erythroid cells, heme regulation is particularly important because it ensures the balanced production of the globin chain components (Tahara et al., 2004a,b). Experiments using knockout mice showed that Flvcr1a and Flvcr1b both play a role in the expansion of committed erythroid progenitors as production of hemoglobin was reduced but only Flvcr1b is indispensable during terminal erythroid differentiation due to a block at the pro-erythroblast stage (Mercurio et al., 2015). RNAi in human lymphoblast K562 cells showed that the coordinated expression of both isoforms controls the cytosolic free heme pool; Flvcr1a deficiency resulted in cytosolic heme accumulation detrimentally effecting cell proliferation but promoted differentiation, while mitochondrial heme accumulation due to Flvcr1b loss was deleterious to both processes. While Flvcr1 has been characterized in vertebrates, a potential role in arthropod heme transport has not been explored.

In 2008, a new family of heme transporters was identified in the nematode *Caenorhabditis elegans*. Rajagopal et al. (2008) performed a genome-wide microarray analysis to identify genes transcriptionally regulated by heme and found F36H1.5 (hrg-4) and its 3 paralogues, R02E12.6 (hrg-1), F36H1.9 (hrg-5), and F36H1.10 (hrg-6) with only two, hrg-1, and hrg-4, found to be highly responsive to heme deficiency and the only genes that were not nematode-specific. White et al. (2013) identified a human/mouse hrg-1 ortholog localized on the macrophage phagolysosomal membranes in mice by immunofluorescence microscopy. Immunohistochemistry showed high levels of hrg-1 in the macrophages present in the tissues responsible for high levels of recycling of heme iron obtained from degraded red blood cells, the spleen, liver and bone marrow of mice and humans. Knockdown of the gene by siRNA in mice bone marrow-derived macrophages resulted in a reduction of the heme regulatory pool while overexpression increased cellular heme availability. Toh et al. (2015) identified and characterized an hrg-1 ortholog in the blood fluke *Schistosoma mansoni* (Smhrg-1), which had a low sequence identity and homology to the *C. elegans* genes hrg-1 and hrg-4 and was found localized via palladium mesoporphyrin IX fluorescence in the vitelline and ovary regions of the females of the species. However, heme transport by Smhrg-1 was only identified in these organs indicating that the heme transport mechanism after digestion to the vitelline and the ovary regions is not due to Smhrg-1 but some other unknown mechanism. A *Leishmania amazonensis* homolog of the *C. elegans* heme transporter hrg-4, LHR1, was identified by Huynh et al. (2012), and was found to localize to both the plasma membrane and the lysosomes as determined by measurement of GFP-LHR1 fusion proteins in cells via confocal laser fluorescence microscopy (Rajagopal et al., 2008; Huynh et al., 2012). Subsequent failure to generate a full knockout of LHR1 was interpreted to indicate the essential nature of this gene to organism survival. Partial knockout and overexpression resulted in reduced heme uptake and increased uptake respectively as measured by zinc mesoporphyrin (ZnMP). Taken together these three observations indicated that LHR1 accounts for the majority of the heme transport activity in *L. amazonensis*. Topology modeling identified four predicted transmembrane domains and cytoplasmic N- and C- termini in *C. elegans* hrg-1 and hrg-4 as well as LHR1 in *L. amazonensis* indicating similar protein structure between hrg family members in these distant eukaryotes (Rajagopal et al., 2008; Huynh et al., 2012). The fact that members of the hrg gene family are present in humans, worms and single-cell protozoan parasites suggests that this gene family is very ancient, however orthologs of this gene family in bloodfeeding arthropods have not been described.

Recently, Lara et al. (2015) identified ATP binding cassette subtype B10 (ABCB10) as a heme transporter in the midgut cells of R. *microplus*. Incubation of midgut cells with Rhodamine 123 (a PgP protein transporter substrate), separately or combined with CsA (an ABC inhibitor) confirmed an ABC transporter was responsible for heme transport after a bloodmeal, while an anti-PgP-1 antibody identified the membrane of digestive vacuoles to be its location in the cells. RNAi of the RmABCB10 transporter in female bloodfed ticks and Zinc protoporphyrin IX (ZnPp) fluorescence showed reduced ZnPp in the hemosomes, but more in the digestive vacuoles, which confirmed RmABCB10 as the transporter characterized above. Lara et al. (2015) confirmed previous reports that identified ABC transporters as key components of detoxification of acaricides by showing that Tin protoporphyrin IX and amitraz transport to the hemosome are increased in the amitraz-resistant Ibirapuã strain when compared to wild type (Pohl et al., 2011, 2012, 2014; Lara et al., 2015; Koh-Tan et al., 2016). Mangia et al. (2016) expanded this to *Ixodes ricinus* by examining expression of ABCB1, ABCB6, ABCB8 and ABCB10 after ivermectin treatment in cultured cells (Mangia et al., 2016). The authors found that only ABCB8 showed changes in expression showing a low-dose stimulation but a high-dose return to control levels after exposure the increasing concentration of ivermectin. These results taken together indicate that ABC transporters transport acaricides in the detoxification pathway of multiple tick species with some family members also transporting heme out of the digestive vacuoles. Like FLVCR and HRG genes, orthologs of ABCB10 have not been described to date in other bloodfeeding arthropods.

Pereira et al. (2007) found evidence of heme transport into the midgut epithelium cells in *Ae. aegypti* and export of its degradation product biglutaminyl-biliverdin back into the lumen for excretion. The authors observed a change in color from red to green during bloodmeal digestion and upon analysis identified it as a bilin pigment. Heme degradation occurs via the cytosolic heme oxygenase reaction, thus heme must enter the midgut epithelium to be degraded and then the biliverdin biproduct must be secreted, although the mechanisms behind the transport of these 2 molecules are unknown. RNA sequencing following heme exposure in *Ae. aegypti* cultured cells identified several potential transport-related proteins that could be involved in this transport mechanism (Bottino-Rojas et al., 2015), however their activity has not been characterized to date. Although, strong evidence of the capacity of heme transport is available for bloodfeeding arthropods, only 1 transporter, RmABCB10 had been characterized to date.

## Heme catabolism

After heme is imported into the cell, a conserved heme degradation pathway is present in many organisms, during which heme is broken down into biliverdin IX α (BV α), iron and CO by heme oxygenase (HO) (for recent reviews, see Wegiel et al., 2014; Wilks and Heinzl, 2014). However, at least two bloodfeeding arthropods, *Aedes aegypti* and *Rhodnius prolixus*, deviate from this pathway as discussed below. In addition, many species of ticks have completely lost the ability to breakdown heme due to absence of key enzymes in the heme degradation pathway in their genomes (Braz et al., 1999; Paiva-Silva et al., 2006; Pereira et al., 2007; Perner et al., 2016). In *Ae. aegypti*, after BV IX, iron and CO are produced, two glutamine residues are added, as determined by electrospray ionization mass spectrometry (ESI-MS) (Pereira et al., 2007). A BV IX product with one glutamine residue was isolated with the final product in reverse phase HPLC indicating that the glutamines were sequentially added instead of simultaneously. This addition to BV IX yields a more soluble product, and was thought to reduce toxicity caused by the accumulation of the heme byproducts in the midgut by allowing for easier excretion (Pereira et al., 2007). The formation of a green pigment identified as biglutaminyl BV IX in the lumen indicates that this byproduct must exit the midgut epithelium cells to be eventually excreted, although the mechanisms behind the transport are unknown. This mechanism is so far unique to *Ae. aegypti*, the pathways utilized by other bloodfeeding dipterans are currently unknown.

**Figure 2:**
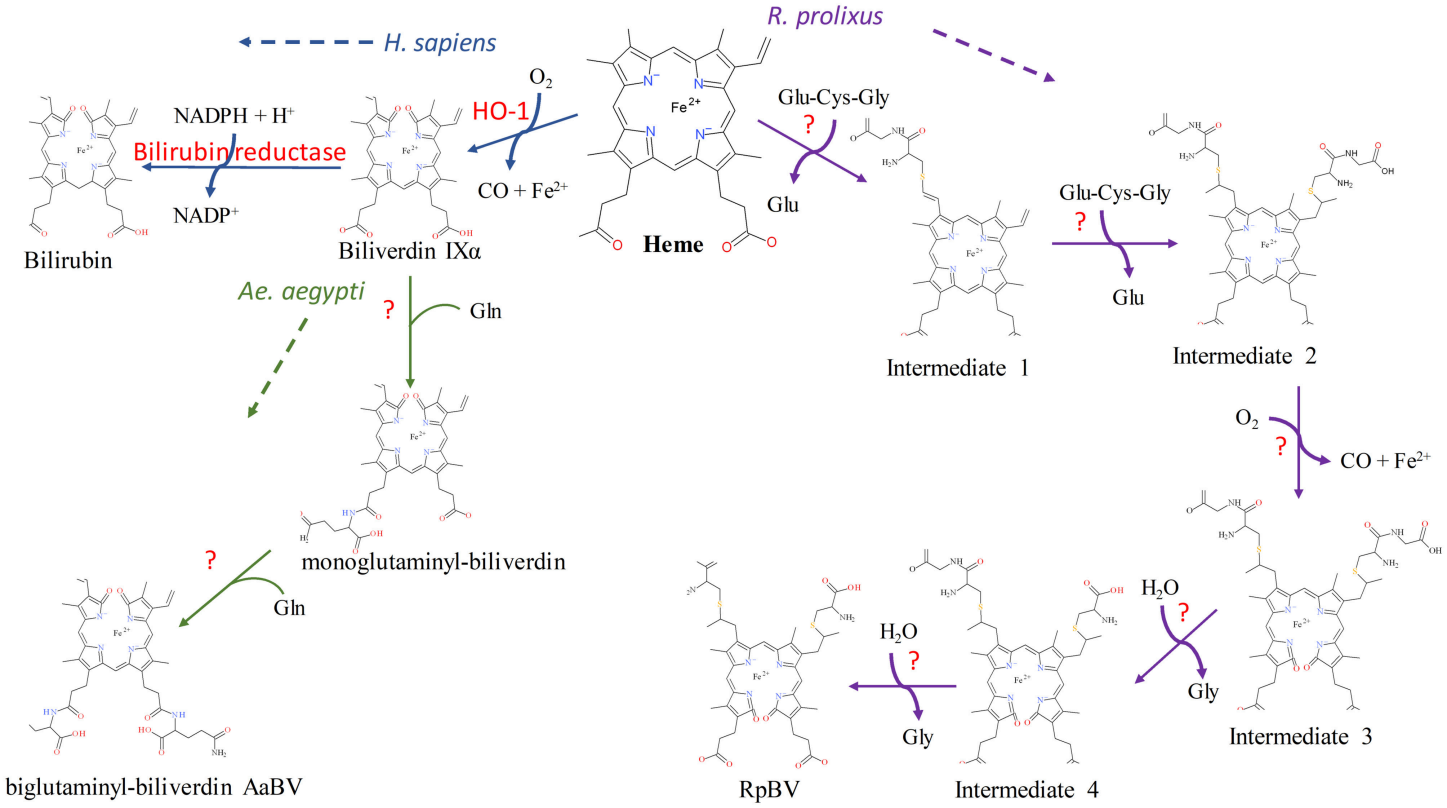
Known heme degradation mechanisms. The arthropods *Ae. aegypti* and *R. prolixus* have alternate heme degradation mechanisms from the shared mechanism of humans, mice and many other organisms. All three mechanisms are detailed above. Enzymes utilized for each reaction are given in red, with unknown enzymes indicated (?). The order in which the modifications are sequentially added to the intermediates in both the *Ae. aegypti* and *R. prolixus* mechanisms is not known, however only one method is detailed here for simplicity. The structural drawings shown here were made using BIOVIA 2017 R2 (Dassault Systemes, San Diego).

In all cases previously reported, heme is broken down by HO prior to any modifications to the porphyrin ring, however the kissing bug contains a unique pathway in which two cysteinylglycine residues are added before oxidative cleavage of the porphyrin ring yielding the end product of dicysteinyl-BV IX 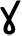 (RpBV) as determined by ESI-MS (Paiva-Silva et al., 2006). While the biological advantage to these added residues is not clear, Paiva-Silva et al. (2006) hypothesized this change in structure is also enacted to increase solubility of the byproducts, easing excretion as confirmed by its elution with a more hydrophilic retention time during reverse-phase HPLC than BV IX α. Also, while RpBV has been identified as the end product, the method of residue addition and the order added are unknown. In mammals, all the degradation products produced by heme play key regulatory roles, for example CO has both anti-inflammatory and anti-apoptotic effects and BV may function as antioxidants, however these roles have not been explored in insects to date (Stocker et al., 1987; Doré and Snyder, 1999; Otterbein et al., 2000; Brouard et al., 2002; Sedlak and Snyder, 2004; Stocker, 2004; Al-Owais et al., 2015). Given at least two variations on heme degradation pathways in arthropods have been described so far, it is likely that there are more not yet discovered. Figure 2 details the proposed mechanisms of the *Ae. aegypti* and *R. prolixus* heme degradation pathways.

In *Drosophila melanogaster*, heme oxygenase expression is necessary for the normal development of tissues. Whole body knockdown of HO in larval and pupal flies results in lethality, evidence of the necessity of heme-iron recycling in tissue development (Cui et al., 2008). Cui et al. (2008) observed the effects of tissue specific HO-knockdown utilizing the GMR-GAL4 driver system to target the eye imaginal disks which resulted in abnormal development of the adult eye tissue. Immunostaining the eye tissue of HO knockdown larvae revealed high concentrations of activated caspase-3, an apoptotic marker, as well as larger than normal iron deposits, both of which were thought to contribute to the rough eye phenotype observed. This observed phenotype was later linked to G1/S arrest of the cell cycle leading to cell death due to increased generation of reactive oxygen species (Ida et al., 2013). Ida et al. (2013) also observed a significant drop in proliferating cells and an increase of DNA damage detected in the eye imaginal disks of the larva after treatment with HO dsRNA. Ida et al. (2013) performed a genomic screen leading to the identification of eight genomic regions that suppressed the observed rough eye phenotype during HO knockdown. This indicates that specific genes on these isolated regions may interact with HO and help counteract its loss during a knockdown event, however further work is needed to isolate specific genes. Damulewicz et al. (2017b) found that *ho* is a clock-controlled gene as it oscillates in expression during the day peaking at the beginning of the light phase and in the middle of the night. One of the reasons behind this pattern of oscillation was due to HO's protection of the retina photoreceptors against ROS-induced degradation brought on during the transition of the night to day phase at the start of UV and white light exposure. DNA damage was reduced when HO was activated by hemin and increased when HO activity was inhibited by Sn PP (Damulewicz et al., 2017a,b). The decline in HO regulated two different canonical clock genes *period* and *clock*, increasing and decreasing respectively. This regulation is potentially mediated through CO as an increase in CO shows the same effect as the increase of HO expression (Damulewicz et al., 2017b). In conclusion, the results of the loss of HO in *Drosophila* tissues and whole body indicate that HO is very important for the successful development of these tissues. However, HO expression and its regulation of DNA damage response and canonical clock genes has not been examined in other insects. This would be particularly important to study in bloodfeeding arthropods as their ingestion of blood yields a much more iron rich diet than flies.

## Iron transport across cell membranes

Iron-responsive genes including those implicated in membrane bound iron transport have been identified in multiple organisms, including arthropods (for recent reviews, see Galay et al., 2015 and Mandilaras et al., 2013). A recent study in *G. morsitans* for example, identified 150 iron-responsive genes, only two of which were previously identified, ferritin heavy chain and mRCK-alpha (Tang and Zhou, 2013b; Dashti et al., 2016). These genes were predicted by computational analysis of UTR regions searching for the characteristic stem loop structures present in iron regulatory element (IRE) regulated genes. Of these genes, 29 had functions potentially related to iron trafficking, including cell envelope, transport and binding proteins that localized in the extracellular environment or the plasma membrane. Genes involved in iron trafficking are particularly important for bloodfeeding arthropods as the blood meal contains a huge influx of iron in the form of transferrin and hemoglobin, 1884.8 ng iron per female on average (Zhou et al., 2007).

Three genes in *D. melanogaster* have been identified as playing a role in membrane bound iron transport, *malvolio* (*mvl*), *zip13*, and *mco1*. Mvl is a homolog of the human natural resistance-associated macrophage proteins (*Nramp*). Both loss and gain-of-function *mvl* mutants in adult flies resulted in altered taste perception, particularly sugar and salt perception (Rodrigues et al., 1995). Folwell et al. (2006) found high expression of *mvl* in adult and larval midgut tissue as well as in the Malpighian tubules, while elucidating its role in iron acquisition at multiple developmental stages and its role in divalent cation reabsorption (Rodrigues et al., 1995; Folwell et al., 2006). Subsequently, an ortholog of *Nramp/mvl* was also characterized in the mosquito *Anopheles albimanus* (Martínez-Barnetche et al., 2007). This group determined that *anaNramp* localized to the head, midgut, Malpighian tubules and ovaries, the highest expression of which was in the Malpighian tubules (Martínez-Barnetche et al., 2007). These authors confirmed the role of *anaNramp* in Fe^2+^ transport by inducing full length cDNA expression in Xenopus embryos and measuring ^59^Fe^2+^ isotope incorporation. An examination of the *anaNramp* 5′ and 3′ UTR sequences indicated that unlike *mvl*, no iron responsive elements (IRE) were present suggesting *anaNramp* may not be regulated by cytoplasmic Fe concentration. Few arthropods have been characterized that contain *h-Nramp* homologs, those that do contain the gene require further study as the regulation method behind their iron transport is unidentified.

Xiao et al. (2014) recently characterized the Drosophila ortholog of the human zinc iron permease 13 (*zip13*), an iron efflux pump that moves iron from the cytosol to the ER/Golgi (Xiao et al., 2014). RNAi knockdown of *dzip13* resulted in iron deficiency in the entire body of the fly with a reduction of about 50% of wild type iron levels except for iron in the cytosol of the gut cells which showed higher than wild type iron levels. On the other hand, *zip13* overexpression in the midgut resulted in increased iron content throughout the body. The authors then examined *ferritin* and *malvolio* as examples of genes involved in iron metabolism that could act as additional indicators of cytosolic iron levels. They found that when *zip13* expression was knocked down, ferritin expression increased and an Mvl expression decreased, with the opposite expression patterns observed when *dZip13* was overexpressed. The overexpression results matched ferritin and Mvl levels observed when larvae were fed an iron-supplemented diet indicating ferritin and Mvl expression levels seem to be a good indicator of cellular iron levels in Drosophila. With the exception of d*zip13* and *malvolio* no other iron transporters have been identified in *Drosophila*. While orthologs of *dzip13* and *malvolio* are present in many bloodfeeding arthropods, involvement in iron transport hasn't been confirmed in any to date, with the exception of *anaNramp* described above.

The multicopper oxidase enzyme family includes oxidases that target different types of substrates including iron, copper, ascorbic acid and bilirubin (Sakurai and Kataoka, 2007). dMCO1, while not an iron transporter, facilitates transport by acting as a ferroxidase at the membrane which oxidizes reactive aqueous ferrous iron (Fe^2+^) to ferric iron (Fe^3+^) in the hemolymph allowing its binding to transferrin and similar iron binding proteins for transport in *D. melanogaster*. Lang et al. (2012) found dMCO1 localized to both the digestive system and Malpighian tubules, specifically the basal surfaces of each using RT-PCR on organ extracts to calculate expression of the transcript, followed by immunostaining tissues to visualize its location (Lang et al., 2012). dMCO1's role in iron homeostasis was confirmed by RNAi knockdown experiments yielding increased longevity when flies fed on high iron food and a decreased iron accumulation in the body. The MCO gene family is conserved in coleopterans, lepidopterans and dipterans (Lang et al., 2012; Liu et al., 2015; Peng et al., 2015; Ye et al., 2015). *An. gambiae* contains 5 putative multicopper oxidases, with AgMCO1 considered the functional ortholog to dMCO1, though experimental confirmation of this is lacking (Gorman et al., 2008). Very few putative MCO enzymes have been characterized to date in bloodfeeding arthropods and those that do contain these enzymes, require further work to identify which substrate they target and whether or not they are involved in iron metabolism.

## Iron chaperones

Metallochaperones facilitate metal ion storage in target proteins via specific protein-protein interactions at their docking surface tuned to recognize these partner proteins (Rosenzweig, 2002). Iron metallochaperones could be particularly important to iron homeostasis as they bind and safely transport ferrous iron around the cell, preventing oxidative damage from occurring (Philpott et al., 2017). Four cytosolic iron metallochaperones were discovered in humans that are thought to aid in ferritin iron loading: human poly(rC)-binding protein 1 (PCBP1) and its paralogues PCBP2, PCBP3, and PCBP4. When either PCBP1 or PCBP2 and ferritin are expressed in yeast cells, the amount of iron loaded into ferritin drastically increased when compared to ferritin expression by itself (Shi et al., 2008; Leidgens et al., 2013). This was confirmed by RNAi knockdown of PCBP1, PCBP2 or both proteins in human cultured cells and observation of the amount of ^55^Fe incorporated into endogenous cytosolic ferritin, which led to similar reductions in iron uptake into ferritin compared to control cells indicating that both proteins are needed independently for efficient delivery of iron to ferritin (Shi et al., 2008; Leidgens et al., 2013). The other two family members, PCBP3 and PCBP4 also showed increased iron loading into ferritin (Leidgens et al., 2013). PCBP2 was also shown to interact with NRAMP2 via its cytoplasmic N-terminal region and with ferroportin, the main ferrous iron exporter, lending further credence to its function as an iron chaperone (Yanatori et al., 2014). Interestingly, HO1 was found to complex with PCBP2 but not any of its paralogues (PCBP1, 3 or 4) or the NADPH-cytochrome P450 reductase (CDR) complex competitively, with PCBP2 affinity to HO1 seeing a significant reduction when in heme loaded cells or when PCBP2 is bound to ferrous iron (Yanatori et al., 2017). While these proteins have been characterized in humans, orthologs proteins have not been characterized in any arthropod species to date. If orthologs of the PCBP family of proteins exist in bloodfeeding arthropods, these could indicate a possible missing link in known mechanisms related to iron homeostasis as they may be involved in ferrous iron transport through the midgut epithelium cells, thus preventing the oxidative damage associated with free ferrous iron in the cell.

## Iron packaging and ferritin shuttling

Free iron can enter the cytosol through direct transport from outside the cell or be released internally following heme catabolism. Fe^2+^ is the reactive soluble form of iron while Fe^3+^ is both unreactive and insoluble. Ferritins chaperone and transport iron preventing large concentrations of cytosolic Fe^2+^ which can easily react with lipids, proteins and other cellular components causing oxidative damage. The Ferritin-like superfamily is present in many organisms including arthropods, with all individual members thought to be evolved from a rubrerythrin-like ancestor which played a role in the defense against reactive oxygen species (Andrews, 2010). Ferritin is typically composed of 24 subunits, which fold to create a large cavity that can store 1,500 + Fe^3+^ molecules as well as smaller amounts of other metals like zinc and magnesium (Gutiérrez et al., 2013). Insects have two ferritin subunits, very similar to vertebrate ferritin subunits, heavy-chain homolog (HCH) and light-chain homolog (LCH) (Pham and Winzerling, 2010). HCH, like dMCO1, also has ferroxidase activity and thus catalyzes the oxidization of Fe^2+^ to Fe^3+^ for storage in ferritin; LCH is involved in iron core formation. In most insects, ferritin contains a signal peptide directing it to the endoplasmic reticulum for translation where it stays until iron loaded ferritin is exported out of the cell via secretory vesicles (Nichol et al., 2002; Dunkov and Georgieva, 2006; Pham and Winzerling, 2010). As ferritin loading occurs in the ER and not the basal surface of the midgut epithelial membrane, dMCO1's ferroxidase activity is not utilized to facilitate loading of the iron into ferritin. Excellent reviews are available covering insect ferritins (Dunkov and Georgieva, 2006; Pham and Winzerling, 2010; Tang and Zhou, 2013b), so they are only mentioned briefly here.

Loss of ferritin following gene knockdown or knockout resulted in growth abnormalities ending in death in *D. melanogaster* (González-Morales et al., 2015). Lack of iron present in embryos either due to knockout of ferritin or lack of maternally derived iron also resulted in serious abnormalities often culminating in death during early development. When midgut specific knockdown was performed in *D. melanogaster*, iron accumulated in the iron cell region while systematic iron deficiency was observed throughout the rest of the body, confirming the importance of ferritin in serving as a transport mechanism (Tang and Zhou, 2013a). However, the exact mechanism of ferritin iron transport to non-intestinal tissues after export out of the midgut epithelium remains unknown, as while labeled ferritin or its substrate iron has been identified in multiple tissues across both *D. melanogaster* and *Ae. aegypti* their entry method into other cellular tissues remains uncharacterized (Zhou et al., 2007; Li, 2010; Tang and Zhou, 2013a).

In mosquitoes and other bloodfeeding arthropods ferritin is particularly important due to the large influx of iron they obtain during a blood meal. In *Ae. aegypti*, both ferritin subunits, HCH and LCH, increase in expression after an iron overload or a blood meal, indicating that ferritin may serve as a cytotoxic protector (Dunkov et al., 2002; Geiser et al., 2003). In mosquitoes, different cell types handle ferritin iron storage in different ways. Geiser et al. (2015) found that CCL-125 *Ae. aegypti* larval epithelial-like cells and 4a3b *An. gambiae* larval hemocyte-like cells experienced high levels of iron uptake using Calcein fluorescence assays upon exposure to high levels of iron (Geiser et al., 2009; González-Morales et al., 2015). However, the inductively coupled plasma mass spectrometry (ICP-MS) they performed showed low levels of cytoplasmic free iron present indicating that upon uptake, iron is immediately bound to proteins like ferritin and secreted (Geiser et al., 2009; González-Morales et al., 2015). When comparing the two cell types examined, Geiser et al. (2015) noted that after packaging of iron into ferritin occurred, the CCL-125 cells exported the ferritin out of the cell while 4a3b cells retained it. They speculated that since hemocyte cells are involved in the immune response, hoarding an essential nutrient like iron is likely done to prevent foreign cells access to it providing a more hostile environment for their growth. This immune response is observed in both *Ae. aegypti* and *Bombyx mori* (silkworm), when ferritin is upregulated during bacterial infection lending evidence that host-bacteria regulatory mechanisms involving ferritin production do exist in these organisms (Geiser et al., 2013; Otho et al., 2016). However, the signaling mechanism which determines when secretion occurs or which cell types secrete iron loaded ferritin or retain it has not been identified to date.

Ticks and insects have evolved different mechanisms to ensure free heme is dealt with during digestion, however they both utilize ferritin to package and transport the molecular iron absorbed from the blood meal (Donohue et al., 2009). Recently, Galay et al. (2013, 2014) characterized two distinct ferritins in the hard tick, *Haemaphysalic longicornis*. Hlfer1 and Hlfer2 both have unique functions: storage of iron in the midgut, and secretion from the midgut for transport to other organs with the subunit composition of each potentially controlling their different functions (Galay et al., 2013). Unlike *hlfer1*, the sequence of *hlfer2* lacks an IRE, indicating that it may not be regulated by cytoplasmic Fe concentration, and contains a signal peptide much like the secretory ferritin characterized by Hajdusek et al. (2009) in the hard tick *Ixodes ricinus* (Hajdusek et al., 2009; Galay et al., 2013). RNAi experiments showed the importance of both ferritins present to successful feeding and reproduction in both *H. longicornis* and *I. ricinus*. While intestinal cell types have been studied extensively, knowledge of the regulation of ferritin subunits in non-intestinal cell types and whether multiple subunits work together in different situations is lacking.

Transferrin is an iron binding glycoprotein that is also conserved between vertebrates and arthropods. Unlike ferritin, transferrin can only carry two molecules of Fe^3+^ at a time. In mammals, transferrin is primarily utilized in iron transport in the blood to bring iron to the erythrocytes to be utilized in heme production (Zhang and Enns, 2009). *D. melanogaster* transferrin and *Ae. aegypti* transferrin 1 were both found to be expressed in larval, pupal and adult stages but not in embryos, with expression in *Ae. aegypti* found mainly in the fat body where it is secreted into the hemolymph with high levels of juvenile hormone acting as a negative regulator of its expression (Yoshiga et al., 1999; Harizanova et al., 2005). A second transferrin gene was described in *Ae. aegypti* with weaker iron binding due to key amino acid mutations in the binding pocket as compared to other members of the transferrin family (Zhou et al., 2009). Expression of both genes differ in the adult female mosquitoes, with *AaTf1* expression highest at 24 h post bloodmeal and *AaTf2* expression highest at 72 h post bloodmeal compared to sugar fed females (Zhou et al., 2009). This difference in expression suggests distinct roles in iron metabolism, however the precise role these genes play remains to be determined. Bacterial infection of *Ae. aegypti* also increases transferrin expression, particularly of *AaTf1*, suggesting that *AaTf1* may play a role in sequestering iron during pathogen infection (Zhou et al., 2009). Other studies have also shown transferrin upregulation in mosquito host response to pathogen infection, particularly *Wuchereria bancrofti*, in *Ae. aegypti* and *C. quinquefasciatus* females (Paily et al., 2007; Magalhaes et al., 2008). An early genome survey by Dunkov and Georgieva (2006) identified members of the transferrin family in several insect species; including four in *An. gambiae* and *Ae. aegypti*. However, little is known about the biological role or specialization of these transferrin genes. In summary, transferrin has been identified as an important regulator of iron homeostasis during blood digestion, larval and pupal development and bacterial infection in arthropods. However, most of the underlying data derives from inferred similarity with vertebrate transferrins. The exact signaling mechanism that activates transferrin expression upon pathogen infection has not been identified nor has the role of transferrin as an iron transporter been extensively studied in bloodfeeding insects.

## Heme/iron signaling

In addition to its role as a nutrient, heme acts as a signaling molecule triggering many biological pathways. In *Ae. aegypti* cultured cells, heme was found to regulate the expression of several hundred transcripts, including those associated with redox stress, metabolism and transport related proteins, suggesting the existence of distinct signaling pathways regulated by heme (Bottino-Rojas et al., 2015). Analysis of these genes found that several immune genes were downregulated in response to heme exposure, indicating that the exposed cells may be more susceptible to immune challenge. This was confirmed both *in vitro* and *in vivo* by introduction of *E. cloacae* to heme incubated cultured cells and by orally challenging heme-fed females with *Serratia marcescens* resulting in diminished expression of immune genes both in cells and the midgut and a 2-fold increase in microbial growth. However, the mechanism by which heme is effects the immune pathways and the molecular mechanisms behind certain induced genes are not currently well understood.

While the role of heme signaling in regulating the immune response of bloodfeeding arthropods is just being appreciated, the role of heme in regulating reproduction in bloodfeeding mosquitoes is well established. In the arthropods *D. melanogaster* and *Ae. aegypti*, heme binding to the nuclear receptor E75 can result in the activation of the steroid hormone 20-hydroxyecdysone (20E), a key component in molting, metamorphosis and vitellogenesis (Segraves, 1994; Thummel, 1996; Kokoza et al., 2001; Martín et al., 2001). Cruz et al. (2012) found that heme was required for mediating 20E action via its stabilization of E75, implying its role as a signaling molecule to indicate the availability of a blood meal for vitellogenesis. E75's ligand binding pocket was characterized by Reinking et al. in *D. melanogaster*, where a single heme molecule was identified as a tightly bound prosthetic group (Reinking et al., 2005). The *Ae. aegypti* ortholog, like its *Drosophila* counterpart, has three isoforms, all three of which are vital to the regulation of multiple genes (Segraves and Woldin, 1993; Pierceall et al., 1999; Cruz et al., 2012). Aicart-Ramos et al. (2012) examined the heme binding of E75 hemoproteins in four different species of insect, *D. melanogaster, B. mori, O. fasciatus* and *B. germanica*. In the first two insects, heme is only tightly bound while in the latter two it is covalently attached (Reinking et al., 2005; Aicart-Ramos et al., 2012). In all four insects, heme is so tightly bound that E75 could not be purified without it. Reinking et al. (2005) also described E75 as a potential heme sensor, because upon treatment of cultured cells with an increased concentration of heme, the cells induced up to 8-fold increase of E75 expression indicating that the expression of E75 is directly proportional to the available heme level. Aicart-Ramos et al. (2012), however disagreed with this definition, stating that heme's tightly bound nature precludes it from acting as a heme sensor, since a heme sensor must be able to reversibly bind heme (for a detailed review of heme sensor proteins see Girvan and Munro, 2013). However, this appears to be a disagreement concerning the definition of a heme sensor, not experimental evidence disproving the evidence collected by Reinking et al. (2005), Aicart-Ramos et al. (2012), and Reinking et al. (2005).

**Figure 3:**
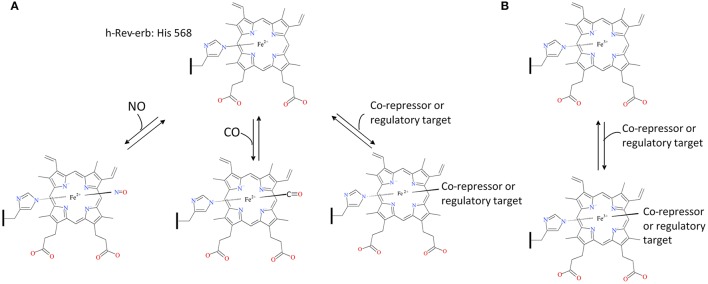
E75-Heme as a cellular oxidative state sensor. The human ortholog of insect E75, h-Rev-erb, acts as a carbon monoxide and nitric oxide sensor when the cell exists in a reducing environment, inhibiting binding of Rev-erb to its co-repressors and regulatory targets **(A)**. Rev-erb's gas sensor ability is not present during a cellular oxidizing environment allowing uninhibited binding to its co-repressors and regulatory targets **(B)**. The structural drawings shown here were made using BIOVIA 2017 R2 (Dassault Systemes, San Diego).

The regulation of E75 by·NO and CO, common in many organisms, occurs when either gas binds the heme-E75 complex, which blocks the heme active site and prevents binding to E75's other regulatory targets (for detailed reviews on NO and CO signaling see Gullotta et al., 2012; Farrugia and Szurszewski, 2014; Jeffrey Man et al., 2014). Therefore, E75 is likely to act as a cellular oxidative state sensor since these gaseous molecules can only bind to ferrous iron not ferric, thus binding would only occur when the cellular state was reduced enough to allow the heme iron to be in its ferrous state (Figure 3). To determine E75's status as a redox sensor, further work must be completed to determine if the cellular environment can actually affect the binding capacity of the protein.

## The role of hemolymph heme and iron-binding proteins: heme, iron and oogenesis in anautogenous arthropods

In addition to the iron carrier proteins, transferrin and ferritin, many arthropods utilize heme/iron carrier proteins which chaperone maternal heme/iron to the developing eggs. This is especially important in ticks, as many species lack key enzymes in the heme synthesis pathway requiring heme to be acquired entirely exogenously (Braz et al., 1999; Perner et al., 2016). Perner et al. (2016) showed that hemoglobin was not necessary for egg production, but was essential for embryo survival in *Ixodes scapularis* (Perner et al., 2016). Female ticks were fed with whole blood or hemoglobin-free serum; eggs were laid by both sets, however only those laid by the bloodfed females hatched and developed normally. Hemoglobin was confirmed as the critical factor by performing rescue experiments with females fed on serum +10, 1, and 0.1% hemoglobin added prior to the rapid engorgement phase which showed that as little as one hundredth of the physiological concentration of hemoglobin was sufficient to rescue tick reproduction. Likewise, Perner et al. (2016) demonstrated that IrCP3 is the major heme-binding protein in *Ixodes ricinus* hemolymph (Perner et al., 2016). Expression profiling over *I. ricinus* developmental stages and tissues revealed that *ircp3* mRNA was consistently up-regulated by bloodfeeding and was predominantly expressed in the trachea-fat body complex, and to a lesser extent, in salivary glands and ovaries of adult females. The authors also confirmed IrCP3 as the most abundant protein in tick hemolymph via SDS PAGE and Western blot analysis. Interestingly, the hemolymph lipoglycoheme-carrier protein from the American dog tick, *Dermacentor variabilis*, was also found to bind heme suggesting that *D. variabilis* carrier protein may function to sequester heme derived from the digestion of the blood meal (Gudderra et al., 2001). Likewise, through spectrophotometric titrations, Maya-Monteiro et al. (2000) found an analogous heme lipoprotein (HeLp) to be a heme-binding protein abundant in the hemolymph of male and female *R. microplus* (To determine if HeLp is involved in extracellular transport, specifically to the ovaries, ^55^Fe-heme-HeLp was injected into the hemocoel of female ticks. Decreased radioactivity was seen in the hemolymph of injected females by 210 min. Most interestingly, by 4 h after hemocoel injection, radioactivity was found associated with the ovaries of female *R. microplus*. However, *R. microplus* females maintained at 4°C after injections lacked clearance of ^55^Fe-heme-HeLp from the hemolymph or incorporation into the ovaries. Therefore, this suggests active metabolism is involved (Maya-Monteiro et al., 2000). Vitellin (VN), the main yolk protein, has also been associated with heme-binding function in *R. microplus* ovaries (Logullo et al., 2002). Through heme-binding assays with purified VN, Logullo et al. (2002) demonstrated that a single VN bound 30 or more heme molecules. Given that both HeLp and vitellin have been ascribed heme-binding functionally and associated with egg development in *R. microplus*, this raises the questions as to whether these two proteins are necessary and sufficient to provide the developing *R. microplus* egg with heme reserves.

Walter-Nuno et al. (2013) found that the *R. prolixus* heme binding protein (RHBP) is an essential transporter of heme to the embryos. RNAi mediated knockdown of RHBP did not alter fecundity, however the resultant eggs were not viable and were white in color. This is in contrast to the dark red embryos oviposited by controls. Taken together, the above studies shed light on the sheer importance of heme-binding and iron-binding proteins in terminal incorporation of heme and iron into the developing eggs of hematophagous arthropods, in particular ticks, which lack the *de novo* heme synthesis pathway. Whether corresponding heme carrier proteins are utilized by bloodfeeding arthropods that maintain the ability to synthesize heme remains unknown.

## Conclusions

Although many arthropods have evolved the ability to feed on blood, relatively little insight into the internal processing of blood meal heme and iron is available. Much of the available information regarding iron processing in arthropods, more specifically insects, is gleaned from *D. melanogaster*. While certain life history traits make *D. melanogaster* a powerful model organism, they do not bloodfeed, highlighting the need to perform direct experimentation on the bloodfeeding arthropods.

**Figure 4:**
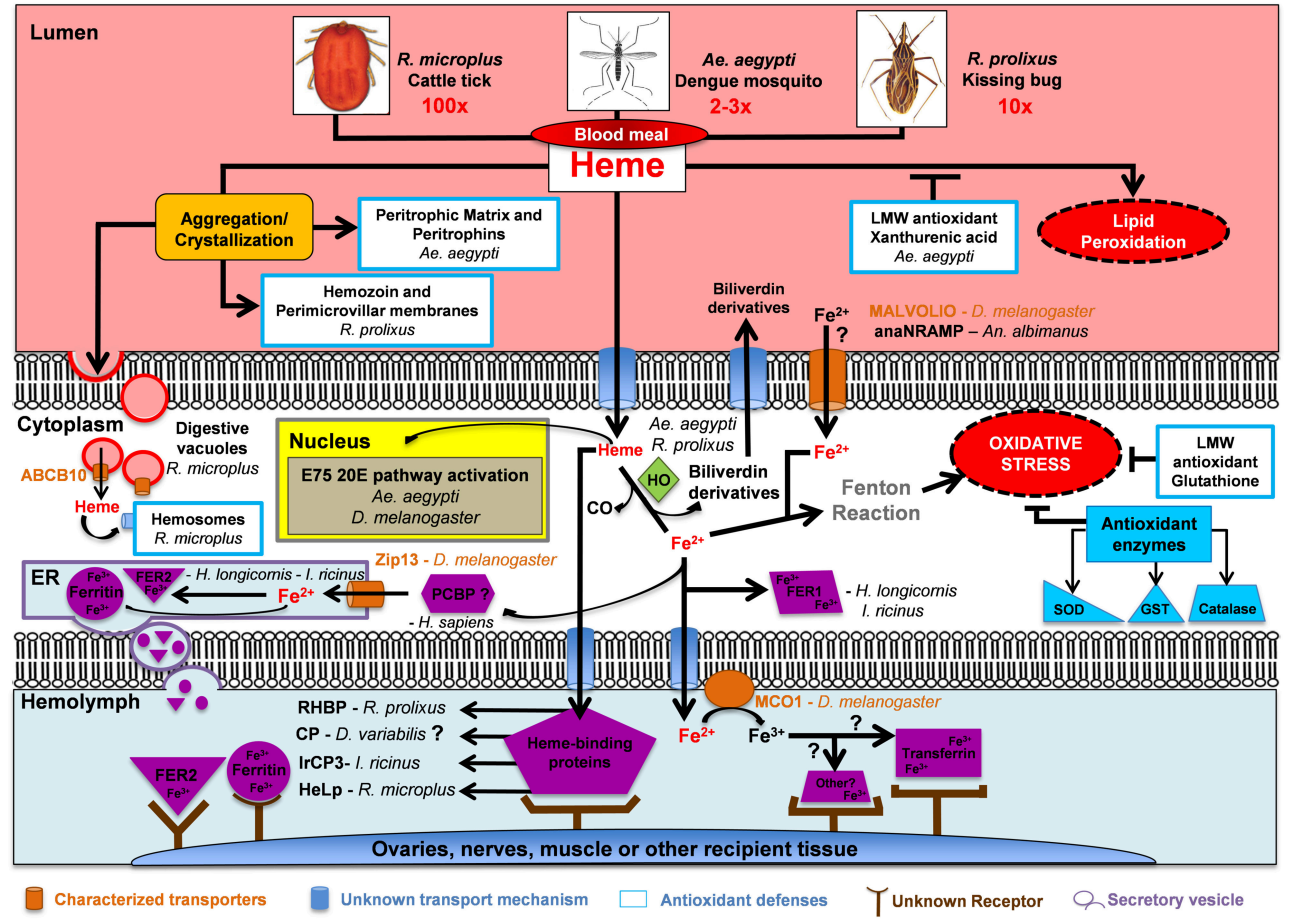
Fate of blood meal heme and iron in selected hematophagous arthropods. Hematophagous arthropods ingest a large amount of blood during a single meal (2-3x, 10x, and 100x their normal body weight for *Ae. aegypti, R. prolixus*, and *R. microplus*, respectively). Hematophagous arthropods deploy a multifactor mechanism to avoid oxidative stress after a blood meal. This multifactor mechanism includes: (1) Aggregation/crystallization of heme for excretion; (2) Transport of heme and iron across plasma membranes; (3) Intracellular degradation of heme by Heme Oxygenase (HO); (4) Enzymatic (shown in dark blue) and non-enzymatic low molecular weight (LMW) antioxidant molecules; and (5) Heme and iron carrier proteins (shown in purple) which deposit these two molecules into ovaries, nerves, muscle or other recipient tissue. Abbreviations: *Anopheles albimanus* natural resistance-associated macrophage protein (anaNRAMP), ATP binding cassette subtype B10 (ABCB10), carrier protein (CP), endoplasmic reticulum (ER), ecdysone-induced protein 75 (E75), Ferritin (Fer), glutathione S-transferase (GST), heme lipoprotein (HeLp), *Ixodes ricinus* carrier protein 3 (IrCP3), multicopper oxidase 1 (MCO1), poly(rC)-binding protein (PCBP), *Rhodnius prolixus* heme binding protein (RHBP), superoxide dismutase (SOD), zinc iron permease 13 (Zip13), 20-hydroxyecdysone (20E).

Through this review, we provide a framework for future studies aimed at obtaining a better understanding as it relates to the fate of heme and iron processing in hematophagous arthropods (Figure 4). With recent advancements in scientific tools such as high-throughput protein and RNA analysis, as well as CRISPR/Cas9, we are optimistic that many questions related to the detoxification, acquisition, transport and ultimate fate of iron/heme in bloodfeeding arthropods can be effectively addressed. Below we have highlighted some key take away points and scientific knowledge gaps that await tackling.

- Given that catalase appears to be a key antioxidant in both midgut and ovarian oxidative stress avoidance for many hematophagous arthropods, further studies targeting this enzyme are needed to assess its potential for disrupting the midgut and ovarian oxidative balance on a larger scale when hematophagous arthropods take a blood meal.
- Heme aggregation/crystalization appears to be a common first line of defense in many hematophagous arthropods. Better understanding of this multifaceted protection playbook for each hematophagous arthropod can provide insight into key targets for breakdown of this system.
- In 2009, Dinglasan et al. set the stage for midgut PM protein composition exploration and discovery utilizing a highly sensitive MS-based approach. The success of this methodology was largely associated with the availability of an artificial protein-free meal. Such protein-free artificial meals for other hematophagous arthropods are needed to allow similar exploration of related and divergent PM structures.
- While peritrophic matrix peritrophins have been identified in many hematophagous arthropods, only two PM peritrophin has been knocked-down using RNAi (APER1 in *An. coluzzii* and peritrophin-1 in *I. scapularis)*. Further studies utilizing reverse genetic tools, such as RNAi and CRISPR/Cas9 are needed to determine the physiological function of PM peritrophins in medically important hematophagous arthropods.
- To date, only one quantitative analysis tracking the fate of blood meal iron in a hematophagous arthropod (*Ae. aegypti*) has been conducted (Zhou et al., 2007). A detailed road map for blood meal iron trafficking in other hematophagous arthropods is needed.
- Heme digestion and utilization regardless of its source, biosynthesis pathway or intake through diet, requires heme to pass across cell membranes. Despite, the characterization of membrane bound heme transport in vertebrates and nematodes, only one transporter has been characterized in a bloodfeeding arthropod to date indicating further work is needed to identify the heme import mechanisms utilized in these organisms.
- Heme catabolism by heme oxygenase 1 to biliverdin IX α, iron and CO is a conserved process in many organisms, however *Ae. aegypti* and *R. prolixus* deviate from this standard and produce alternate bilin pigments, thus there may be more deviations from the known standard yet to be discovered in other bloodfeeding arthropods.
- Heme oxygenase was shown to be essential to *D. melanogaster* tissue development as it regulates the DNA damage response and specific canonical clock gene expression. However, heme oxygenase's role in these two pathways has not been explored in bloodfeeding arthropods despite their diet consisting of a much more iron rich diet than flies.
- In *D. melanogaster*, three genes have been identified as membrane bound iron transporters or facilitators of this process, *mvl, zip13* and *mco1*. While orthologs of both *mvl* and *zip13* do exist in bloodfeeding arthropods, their involvement in iron transport has yet be examined in any to date, with the exception of *anaNramp*.
- The human poly(rC)-binding proteins (PCBP1-4) facilitate iron loading of ferritin. Homologues of these proteins in bloodfeeding arthropods could represent a missing link in iron homeostasis in these organisms.
- While ferritin or its substrate, dietary iron, has been localized in multiple tissues in both *D. melanogaster* and *Ae. aegypti* the mode of entry and recovery of iron from ferritin into cells and tissues remains uncharacterized.
- Transferrin, another conserved iron binding transport protein between vertebrates and arthropods, is found in many bloodfeeding arthropods. While transferrins have been shown to be upregulated after a bloodmeal or during pathogen infection, little is known about the biological role or specialization of these transferrin genes.
- Hemolymph heme-binding proteins (RHBP, HeLp, and IrCP3) allow for proper transport of heme and iron to developing eggs. However, further studies are needed to determine if orthologs for these proteins exist in other hematophagous arthropods and their role in proper shuttling of heme to ovaries. Loss of any heme and iron shuttling processes can decrease egg viability, and ultimately be utilized in novel mosquito population control techniques.

## Author contributions

SW, HE, and ZA conceived the manuscript and edited the manuscript into its final form; SW and HE drafted the initial manuscript.

### Conflict of interest statement

The authors declare that the research was conducted in the absence of any commercial or financial relationships that could be construed as a potential conflict of interest.
